# Establishment of an immortalized human endometrial stromal cell line with functional responses to ovarian stimuli

**DOI:** 10.1186/1477-7827-9-104

**Published:** 2011-08-01

**Authors:** Munehiro Yuhki, Takashi Kajitani, Takakazu Mizuno, Yuko Aoki, Tetsuo Maruyama

**Affiliations:** 1 Kamakura Research Laboratories, Chugai Pharmaceutical Co., Ltd., 200 Kajiwara, Kamakura, Kanagawa 247-8530, Japan; 2 Department of Obstetrics and Gynecology, Keio University School of Medicine, Tokyo 160-8582, Japan

## Abstract

Studies on the mechanisms of decidualization and endometriosis are often hampered by lack of primary endometrial cells. To facilitate in vitro studies, we established a human endometrial stromal cell line, KC02-44D, immortalized with human telomerase reverse transcriptase. Upon exposure to ovarian stimuli, KC02-44D cells showed similar cytoskeletal marker or gene expression and biochemical phenotype to primary endometrial stromal cells. KC02-44D would be useful for studies of human endometrial function and its associated pathologies.

## Background

Decidualization is the process of differentiation of the endometrium, the mucosa lining the uterine lumen. In endometrial decidualization, estrogen receptor (ER) and progesterone receptor (PR) expressions strictly regulate the tissue responsiveness to cognate ligands. Estrogen can autoregulate ER expression, and PR is a classical estrogen target [1-3]. In humans, decidualization occurs independently of a blastocyst signal during the second half of the menstrual cycle and is controlled as a progesterone- and 3',5'-cyclic adenosine monophosphate (cAMP)-dependent event. Its initiation requires elevated intracellular cAMP levels and sustained activation of the protein kinase A (PKA) pathway; progestin and cAMP synergistically induce decidualization in primary cultured human endometrial stromal cells (ESCs) [4]. Decidual cells exhibit morphological changes and abundant secretion of decidualization enhancers such as prolactin and insulin-like growth factor-binding protein-1 (IGFBP-1) [4].

Endometriosis is the growth of endometrial epithelial and stromal cells at ectopic sites outside the uterine cavity. Interleukin (IL)-1β, a proinflammatory cytokine, is considered to play an important role in the pathogenesis of endometriosis [5,6]. However, the relationship between IL-1β and abnormal growth of endometrial cells is not clearly understood.

For investigations on the mechanisms of decidualization and endometriosis, cultured human ESCs are desirable tools; however, the applicability of these cells is largely restricted by the long cell-culture time and difficulty in maintaining the primary ESC phenotype. Several attempts have been made to immortalize human ESCs by oncogenic transformation [7] or prolongation of cell division by introducing human telomerase reverse transcriptase (hTERT) [8-11]. However, the synergistic effect of progestin and cAMP on the decidualization capacity and the induction of the PR by estradiol (E2) have not been fully reproduced in these cell lines. In this study, we established an hTERT-immortalized ESC line, KC02-44D, to facilitate in vitro studies. KC02-44D cells responded in a similar manner to primary human ESCs, showing the synergistic induction of decidualization by progestin and cAMP, and PR upregulation by E2.

## Methods

### Human tissue samples

Endometrial tissue was obtained from the uterus of a 45-year-old woman who had undergone total hysterectomy because of leiomyoma, after her written informed consent and Keio University Hospital approval. Primary human ESCs were purified as described previously [12]. In brief, the tissue specimens were washed in Dulbecco's modified Eagle's medium (DMEM; Sigma-Aldrich, St. Louis, MO) and minced with scissors into pieces less than 1 mm^3 ^in size. The tissues were then gently agitated in tubes for 2 h at 37°C in DMEM with 0.2% (wt/vol) collagenase (Wako Pure Chemical Industries, Osaka, Japan), 0.05% DNase I (Life Technologies, Inc., Gaithersburg, MD), 10% fetal bovine serum (FBS), and 1% antibiotic-antimycotic mixture (Life Technologies, Grand Island, NY). After enzymatic digestion, cell clumps were dispersed by pipetting. Most of the digested human ESCs, presenting as single cells or small aggregates, were filtered sequentially through a 70 μm cell-strainer nylon filter (Falcon 2350; BD Biosciences, Franklin Lakes, NJ) to remove gland cells, layered onto Ficoll-Paque (GE Healthcare Life Sciences, Uppsala, Sweden), and centrifuged at 500 × *g *for 15 min at 4°C to remove erythrocytes. The isolated human ESCs were collected from the Ficoll interface and resuspended in DMEM with 10% FBS and 1% antibiotic-antimycotic mixture.

### Recombinant lentivirus preparations

Recombinant lentiviruses were prepared using the ViraPower Lentiviral Expression System (Life Technologies), according to the manufacturer's instructions. Full-length cDNA of human telomerase reverse transcriptase isoform 1 (hTERT; GenBank accession number NM_003219) was amplified by PCR from the total RNA of a colon cancer cell line. The first-strand DNA was synthesized using hTERT 3'-noncoding primer 5'-TGACAGGGCTGCTGGTGTCTG and ReverTra Ace reverse transcriptase system (Toyobo, Tokyo, Japan). The cDNA of the *N*-terminus half of hTERT (1-2304) was amplified using forward primer 5'-CACCATGCCGCGCGCTCCCCGCTGCCGA and reverse primer 5'-GCCTTCTGGACCACGGCATACCGA, and that of the *C*-terminus half (1977-3399) was amplified using forward primer 5'-CACCGGCACTGTTCAGCGTGCTCAACTACGAG and reverse primer 5'-TCAGTCCAGGATGGTCTTGAAGTC. Each cDNA was ligated to the pLenti6/V5-D TOPO vector. Kozak sequences were introduced according to the instruction manual. The fragment between *Spe*I (in the vector) and *Eco*RV sites from the *N*-terminus half was then recloned in the same sites of the *C*-terminus half to obtain the full-length hTERT plasmid (pLenti6-hTERT). The DNA sequence of pLenti6-hTERT was completely confirmed by DNA sequence analysis. pLenti6-hTERT was then transfected to near-confluent 293FT cells (Life Technologies) with helper plasmids (pLP1, pLP2, and pLP/VSVG) to produce a lentivirus encoding hTERT (LtV-hTERT). After 2 days of incubation at 37°C, the conditioned mediums of the lentivirus-producing 293FT cells were harvested and centrifuged at 3000 rpm for 15 min at 4°C. The supernatants were separated into aliquots and stored at -80°C until use.

### Immortalization of human ESCs

For immortalization, human ESCs were infected with LtV-hTERT in the presence of polybrene (6 mg/ml, Sigma). The cells were grown in phenol red-free DMEM supplemented with 10% dextran-coated charcoal-treated FBS (DCC-FBS; Life Technologies) in the presence of 10 nM 17-b-estradiol (E2; E8875, Sigma) for 7-8 days until uninfected cells died in the presence of 2 mg/ml blasticidine (Life Technologies). Then, the cells were trypsinized and limiting-diluted in 96-well plates. Immortalized clones were obtained in the presence of E2 under selection with blasticidine. The obtained clones were maintained in DMEM supplemented with 10% FBS, 100 U/ml penicillin, 100 mg/ml streptomycin, and 2 mg/ml blasticidine at 37°C under 5% CO_2 _in a humidified incubator. The clones were tested for marker expression and response to ovarian steroids and cAMP, as described below. Finally, KC02-44D was selected as the most responsive clone to the ovarian stimuli. We did not obtain any clones responsive to these stimuli when primary human ESCs were cultured in the absence of E2.

### In vitro decidualization and cytokine assay

KC02-44D cells were precultured in phenol red-free DMEM supplemented with 10% DCC-FBS, 100 U/ml penicillin, and 100 mg/ml streptomycin at 37°C over 4 days. They were then stimulated with or without 10 nM E2, 1 μM 6α-methyl-17α-hydroxy-progesterone acetate (MPA; M-1629, Sigma), and/or 0.125 mM dibutyryl cAMP (D0627, Sigma) in combination with the indicated concentrations of cytokines for different time periods according to the experimental procedures. Cell images were acquired using a phase-contrast microscope (Nikon). In the IL-1β experiment, KC02-44D cells were treated with 1.0 ng/ml IL-1β (201-LB-005, R&D Systems, Minneapolis, MN) for 3-7 days, as described in the figure legends.

### Immunocytochemistry

KC02-44D cells were cultured in 96-well tissue culture plates and characterized by immunofluorescence staining. The cells were fixed with 4% paraformaldehyde (PFA; Wako Pure Chemical Industries) solution for 30 min at room temperature. After washing and permeabilization with phosphate-buffered saline (PBS) containing 0.1% Triton X-100, the cells were blocked with PBS-5% FBS at 4°C. The fixed cells were incubated in PBS-10% FBS containing Cy3-labeled mouse anti-vimentin (C9080, 1:200; Sigma), mouse anti-CD10 (clone SS2/36, 1:25; Dako, Glostrup, Denmark), mouse anti-CD13 (clone WM-47, 1:50; Dako), or mouse anti-cytokeratin (clone MNF116, 1:50; Dako) antibody. After washing in PBS, the cells were incubated with Alexa Fluor 488-labeled anti-mouse secondary antibody (1:500; Molecular Probes, Eugene, OR), washed again, and examined for each marker expression under a fluorescence microscope (BZ-8000; Keyence, Osaka, Japan).

### Western blot analysis

Total proteins from KC02-44D cells were extracted with Cell Lysis Buffer (Cell Signaling Technology, Beverly, MA) containing 1 mM phenylmethanesulfonyl fluoride (Sigma) on ice. The lysate was scraped and sonicated for 5 s by using an ultrasonic disruptor at output 1 (Tomy Digital Biology, Tokyo, Japan). The sonicate was centrifuged at 15,000 rpm for 10 min at 4°C, and the protein contents of the supernatant were measured using the Bradford protein assay (Bio-Rad, Hercules, CA). After the addition of lithium dodecyl sulfate sample buffer (Life Technologies, Grand Island, NY) containing 2-mercaptoethanol (Sigma), the samples were reduced at 70°C for 10 min. Equal amounts of total proteins were then electrophoresed on 4-12% NuPAGE Bis-Tris gels (Life Technologies, Grand Island, NY) and transferred onto polyvinylidene difluoride membranes (Immobilon-P; Millipore, Bedford, MA), followed by blocking with 5% nonfat dry milk in Tris-buffered saline containing 0.1% Tween-20 (TBS-T). The membranes were then immunoblotted with the primary antibody against the PR (clone PgR636, 1:800, mouse monoclonal; Dako), IGFBP-1 (06-106, 1:1000, rabbit polyclonal; Upstate, Millipore), or β-actin (1:3000, goat polyclonal; Santa Cruz Biotechnology, Santa Cruz, CA) in TBS-T for 1 h at room temperature. After washing thrice with TBS-T, the membranes were further incubated with horseradish peroxidase-conjugated anti-mouse, anti-rabbit (1:10,000; GE Healthcare, Buckinghamshire, UK) or anti-goat (1:5000; Jackson ImmunoResearch Laboratories, West Grove, PA) secondary antibody in TBS-T for 1 h at room temperature. After washing thrice with TBS-T again, proteins were detected with ECL Plus reagent (GE Healthcare) and visualized with LumiImager (Roche Diagnostics, Penzberg, Germany).

### Enzyme-linked immunosorbent assay (ELISA)

KC02-44D cells were plated at a density of 2 × 10^5 ^cells per well in 6-well plates. Two days later, the medium was changed and the cells were stimulated with E2, MPA, and dibutyryl cAMP alone or in combination at the indicated concentrations. The supernatants were collected 6 or 7 days later, and the amounts of IGFBP-1, PGE2, and IL-8 proteins were measured using ELISA kits for IGFBP-1 (DY871, R&D Systems), prostaglandin E2 (PGE2; KGE004, R&D Systems), and IL-8 (GE Healthcare), respectively. Each treatment was performed in triplicate wells.

### Reverse transcription polymerase chain reaction (RT-PCR)

Total RNA was extracted from cultured KC02-44D cells by using an RNeasy mini kit (Qiagen, Valencia, CA). RT-PCR was carried out with 200 ng total cellular RNA by using a One-Step RT-PCR kit (Qiagen). The primers used for amplification were as follows: human estrogen receptor-alpha (ERα), forward primer 5'-CAGGGGTGAAGTGGGGTCTGCTG-3' and reverse primer 5'-ATGCGGAACCGAGATGATGTAGC-3'; cyclooxygenase-2 (COX-2), forward primer 5'-TGTCTTGACATCCAGATCAC-3' and reverse primer 5'-ACATCATGTTTGAGCCCTGG-3'; β-actin, forward primer 5'-CCCAGGCACCAGGGCGTGATC-3' and reverse primer 5'-TCAAACATGATCTGGGTCAT-3'. Preliminary experiments determined the optimum PCR cycle number within the linear range of amplification for each gene being measured. After PCR amplification, the samples (15 μl aliquots) were electrophoresed in 2% agarose gels, followed by photographic recording of ethidium bromide-stained gels with FAS-III MINI (Toyobo). All data from the RT-PCR analysis represent the results of three independent experiments.

### Cell-proliferation assay

Cell proliferation was determined using a Click-iT EdU Alexa Fluor Imaging kit (Life Technologies, Grand Island, NY), a modified 5-bromo-2'-deoxyuridine (BrdU) system to detect DNA replication. In brief, KC02-44D cells (1.0-1.5 × 10^3^) were seeded onto 96-well culture plates with phenol red-free DMEM containing 10% DCC-FBS and stimulated with cytokines 48 h later. EdU (5-ethynyl-2'-deoxyuridine) was added to a final concentration of 10 μM in the cell-culture medium and the cells were incubated for 24 h at 37°C. After fixation with 4% PFA and permeabilization with 0.1% Triton X-100 in PBS, the cells were treated with the reaction cocktail containing Alexa Fluor 488 azide. The EdU intensity was measured using an ArrayScan VTi HCS reader (Thermo Scientific Cellomics, Pittsburg, PA). The colocalization bioapplication protocol was used to acquire images with the appropriate filter sets in two separate fluorescence channels: channel 1, Hoechst 33342 (nuclear reference and object identification); channel 2, EdU labeled with Alexa Fluor 488 azide (identification of DNA-replicating S-phase cells in the cell cycle). The cells were imaged with a 20 × objective and 0.63 numerical aperture with exposure times of 0.2 s (Hoechst 33342) and 0.1 s (Alexa Fluor 488 azide). The images were collected as nine fields per well, and the values were expressed as the mean average fluorescence intensity.

### Statistical analysis

All quantitative experiments were conducted at least three times each in triplicate wells. The results were then analyzed using ANOVA and Tukey's test. The data shown in figures was the Mean ± SEM for triplicate wells from one representative experiment.

## Results

### Characterization of KC02-44D cells

#### Immunocytochemical analysis

**Figure 1:**
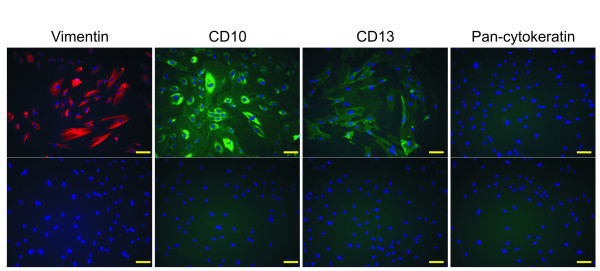
**Expression of marker proteins in KC02-44D cells**. PFA-fixed KC02-44D cells at passage number 10 were stained with antibodies against vimentin (red) as well as CD10, CD13, and cytokeratin (green, with Hoechst blue for nuclear staining). No signal was detected in the negative controls (no antibody; lower panels). Scale bars = 100 μm.

To assess specific antigen marker expression, KC02-44D cells were fixed, permeabilized, and immunostained with antibodies to endometrial stromal marker proteins. The cells were positively stained with the stromal cell markers vimentin, CD10, or CD13, but not with the epithelial cell marker cytokeratin (Figure 1).

#### Responses to ovarian steroids and cAMP

**Figure 2:**
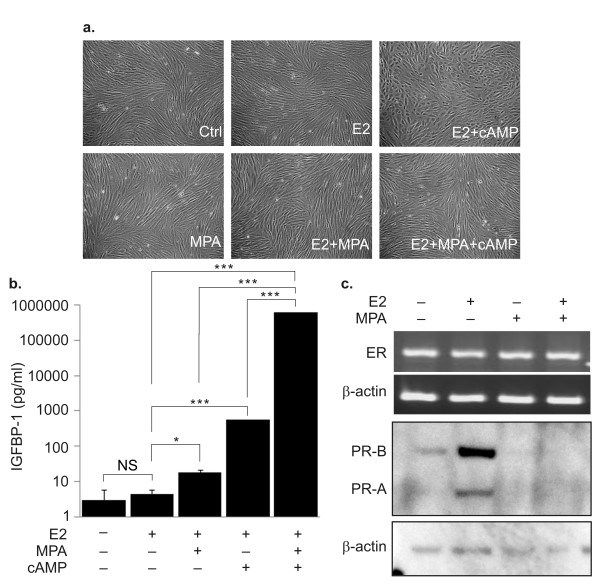
**Induction of decidualization in KC02-44D cells**. KC02-44D cells were treated with the indicated combinations of 10^-8 ^M E2, 10^-6 ^M MPA, and 0.125 mM dibutyryl cAMP for 6 days to assess their decidualization capacity. (a) Phase-contrast microphotographs of living KC02-44D cells at passage number 17 (scale bars = 100 μm). (b) Expression of decidualization marker IGFBP-1 in KC02-44D cells at passage number 15 (ELISA). The data represent the mean ± SEM of triplicate measurements. *P < 0.05 and ***P < 0.001 by Tukey's test. (c) Expression of the ER (RT-PCR analysis) and PR (Western blot analysis) in KC02-44D cells at passage numbers 15 and 16. β-Actin was used for normalization.

The decidualization capacity of KC02-44D cells against ovarian steroids and cAMP was next examined. Decidualized stromal cells are known to change into a round morphology upon treatment with cAMP and a spindle-shaped morphology upon treatment with MPA [9,10]. When KC02-44D cells precultured in the E2-free medium were treated with E2, MPA, and dibutyryl cAMP for 6 days, they transformed into a decidualized morphology (Figure 2a).

KC02-44D cells were also tested for their response to ovarian steroids and dibutyryl cAMP to induce IGFBP-1, a decidualization marker [4]. MPA or dibutyryl cAMP significantly but very weakly induced IGFBP-1 expression in the presence of E2. However, the protein expression was greatly induced by the combination of MPA, dibutyryl cAMP, and E2 (Figure 2b), consistent with the findings of a previous study on primary human ESCs [13]. Prolactin, another decidualization marker, showed similar responses to IGFBP-1 (See Additional file 1 Figure S1). KC02-44D cells maintain their response to estradiol, progestin, and cAMP even at high passage numbers (See Additional file 2 Figure S2).

We then assessed PR expression under stimulation by ovarian steroids. Exposure of KC02-44D cells to E2 strongly upregulated the expression of both isoforms (A and B) of PR, a target gene of ERα in the endometrium [14-18]. The E2-induced PR expression was significantly downregulated after stimulation with MPA (Figure 2c), consistent with reports on the endometrial decidualization phase [7,13,19]. Therefore, KC02-44D cells were considered to possess ESC characteristics, displaying similar biochemical properties as well as cytoskeletal marker and gene expression patterns to primary ESCs, without any structural abnormalities.

### Responses of KC02-44D cells to IL-1β

#### Regulation of inflammatory responses by IL-1β

**Figure 3:**
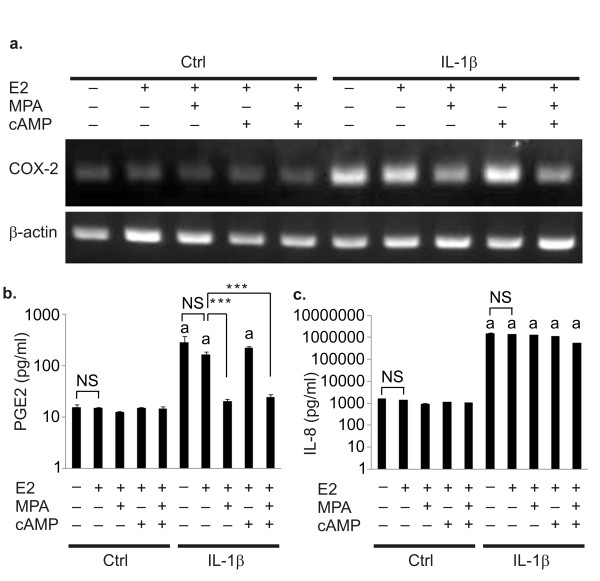
**Inflammatory responses of KC02-44D cells to IL-1β**. KC02-44D cells were cultured with the indicated combinations of 10^-8 ^M E2, 10^-6 ^M MPA, and 0.125 mM dibutyryl cAMP in the absence or presence of IL-1β (1.0 ng/ml) for 7 days to observe their inflammatory responses. (a) Expression of COX-2 in KC02-44D cells at passage number 14 (RT-PCR analysis). β-Actin mRNA was used for normalization. (b) Expression of PGE2 in KC02-44D cells at passage number 16 (ELISA). (c) Expression of IL-8 in KC02-44D cells at passage number 19 (ELISA). The data represent the mean ± SEM of triplicate measurements. ***P < 0.001 by Tukey's test. Letter a indicate differences between the control and IL-1β for each stimulate: p < 0.001.

Pro-inflammatory cytokine concentrations in peritoneal fluid increase in women with endometriosis [20-22], and IL-1β is suggested to play an important role in the pathogenesis of endometriosis [5,6]. Therefore, we investigated the effect of IL-1β on the inflammatory responses in KC02-44D cells. When the cells were treated with IL-1β, COX-2 gene expression was dramatically enhanced (Figure 3a), and PGE2 and IL-8 secretion was induced (Figure 3b and 3c). COX-2 and PGE2 induction was canceled by the addition of MPA. In contrast, IL-8 induction by IL-1β could not be suppressed by MPA, suggesting PR-independent regulation. The vehicle control yielded the same result as E2 (Figure 3b and 3c).

#### Downregulation of decidualization capacity by IL-1β

**Figure 4:**
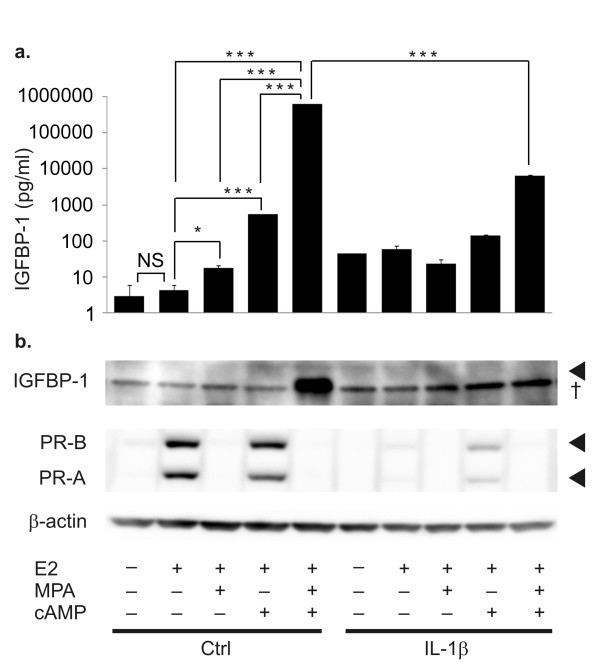
**IL-1β-induced downregulation of the decidualization capacity of KC02-44D cells**. KC02-44D cells were pretreated with IL-1β (1.0 ng/ml) for 4 days and then stimulated with the indicated combinations of 10^-8 ^M E2, 10^-6 ^M MPA, and 0.125 mM dibutyryl cAMP for 7 days. (a) Expression of decidualization marker IGFBP-1 in KC02-44D cells at passage number 19 (ELISA). The data represent the mean ± SEM of triplicate measurements. *P < 0.05, *P < 0.01 and ***P < 0.001 by Tukey's test. (b) Expression of IGFBP-1 and the PR in KC02-44D cells at passage number 16 (Western blot analysis). β-Actin was used for normalization. † = nonspecific band.

Reportedly, stromal cells from endometriotic lesions and the endometrium in women with endometriosis show reduced decidualization capacity [23], and IL-1β inhibits cAMP-mediated decidualization in primary human ESCs [24-26]. Therefore, we tested whether IL-1β can reduce the decidualization capacity of KC02-44D cells. IL-1β drastically inhibited MPA- and dibutyryl cAMP-induced IGFBP-1 expression (Figure 4a); moreover, the E2-induced PR expression was significantly downregulated after incubation with IL-1β (Figure 4b). The vehicle control yielded the same result as E2 for IGFBP-1expression (Figure 4a and 4b).

#### Counteraction of ovarian steroid- and cAMP-induced growth arrest by IL-1β

**Figure 5:**
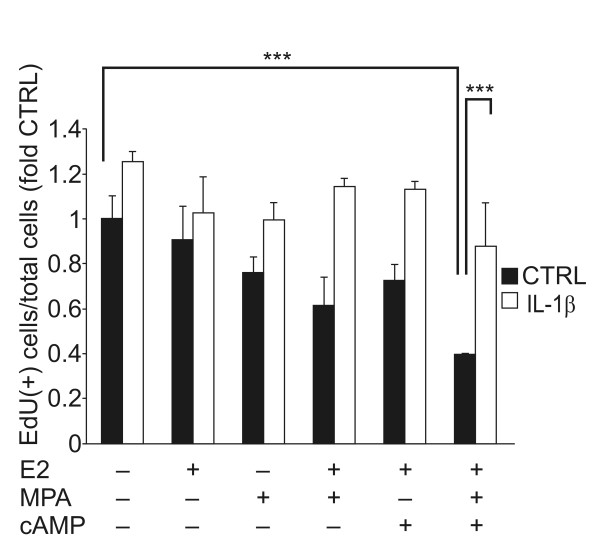
**Counteraction of decidualization stimuli-induced cell growth arrest by IL-1β**. EdU assay was performed with KC02-44D cells at passage number 15 pretreated with IL-1β (1.0 ng/ml) for 3 days and then stimulated with the indicated combinations of 10^-8 ^M E2, 10^-6 ^M MPA, and 0.125 mM dibutyryl cAMP for 24 h. The EdU intensity was measured by an ArrayScan reader. The data represent the mean ± SEM of triplicate measurements. ***P < 0.001 by Tukey's test.

We used the EdU assay to assess whether IL-1β can counteract MPA- and dibutyryl cAMP-induced growth arrest of KC02-44D cells. In the absence of the ovarian steroids and dibutyryl cAMP, IL-1β slightly enhanced EdU incorporation to DNA, indicating the induction of cell proliferation (Figure 5). Along with the decidualization mediated by MPA and/or dibutyryl cAMP, the cell growth was significantly inhibited, observed as a reduction in the number of EdU-labeled S-phase cells (Figure 5, CTRL). In contrast, IL-1β treatment counteracted cell growth arrest even in the presence of MPA and/or dibutyryl cAMP (Figure 5, IL-1β), indicating the inhibition of the decidualization capacity of KC02-44D cells.

## Discussion

We established the KC02-44D cell line and found similar characteristics to primary human ESCs, showing the synergistic induction of decidualization by progestin and cAMP. Moreover, our cell line showed upregulation of PR expression upon E2 stimulation, which is critical for the progestin-dependent decidualization capacity of ESCs. According to reports of immortalized cell lines, a simian virus 40 large T antigen (SV40T)-immortalized ESC line did not respond to E2 stimulation for PR expression and prostaglandin production, possibly by nonfunctional ERα [7], and another temperature-sensitive SV40T-immortalized ESC line showed an adequate decidualization phenotype only with cAMP stimulation, even in the absence of progestin [11]. A recent hTERT-immortalized cell line showed a robust response to progestin stimulation, but the synergistic effect of cAMP in this cell line was not examined [9]. Another hTERT-immortalized line showed progestin and cAMP synergy, but in contrast to our clone, PR induction by E2 was not noted in this cell line [10]. The reasons for these discrepancies are not clear, but the differences in human ESC sources or E2-supplemented culture conditions for clone selection might affect the ability of each cell line.

Human ESCs respond to IL-1β through induction of COX-2 expression and PGE2 production. In primary human ESCs, COX-2 mRNA is induced by IL-1β in a p38 mitogen-activated protein kinase (p38MAPK)-dependent manner and this induction is diminished by progesterone or 8-bromo-cAMP through p38MAPK inhibition [27], which is consistent with the results regarding the inflammatory responses of KC02-44D cells. Further, we observed induction of IL-8 production upon IL-1β stimulation. However, progestin and/or cAMP seemed to have no inhibitory effect on IL-8 expression. A previous study using endometriotic tissues showed a similar observation, indicating the menstrual cycle phase-independent expression of IL-8 and progestin resistance of IL-1β-induced IL-8 production [28]. However, the mechanism of progestin resistance of IL-8 induction is currently unclear; a PR-dependent mechanism might be responsible. At least distinct responses were observed for COX-2 and IL-8 induction by IL-1β in our cell line.

In the menstrual cycle, estrogen induces PR expression in the proliferative phase, and a high level of PR expression is observed in the late proliferative phase; this expression is downregulated according to the elevation in the serum progesterone level, followed by the initiation of decidualization in the secretory phase [29,30]. We have shown the regulation of PR expression in this study. E2 strongly upregulated the expression of the PR, and the E2-induced PR expression was significantly downregulated after the stimulation with MPA (Figure 2c). Compared with paired ectopic endometrial specimens, a drastic loss of PR-B expression has been observed in endometriotic tissues, resulting in progesterone resistance in endometriosis [31]. IL-1β-induced inhibition of the decidualization capacity [24-26], activation of cell proliferation, and downregulation of PR-B expression have been observed in primary ESCs [32]. We confirmed the effects of IL-1β on these responses in KC02-44D cells.

## Conclusions

We established an immortalized human ESC line, KC02-44D, by introducing the hTERT gene into primary ESCs. This clone showed characteristics such as decidualization capacity and responses to IL-1β similar to primary human ESCs. The KC02-44D cell line would be useful to investigate the mechanisms of normal and pathological human reproductive processes.

## List of abbreviations used

BrdU: 5-bromo-2'-deoxyuridine; cAMP: 3',5'-cyclic adenosine monophosphate; COX-2: cyclooxygenase-2; DCC-FBS: dextran-coated charcoal-treated fetal bovine serum; DMEM: Dulbecco's modified Eagle's medium; E2: estradiol; EdU: 5-ethynyl-2'-deoxyuridine; ELISA: enzyme-linked immunosorbent assay; ESC: endometrial stromal cell; ERα: human estrogen receptor-alpha; hTERT: human telomerase reverse transcriptase; IGFBP-1: insulin-like growth factor-binding protein-1; IL: interleukin; LtV-hTERT: a lentivirus encoding hTERT; MPA: 6α-methyl-17α-hydroxy-progesterone acetate; PBS: phosphate-buffered saline; PFA: paraformaldehyde; PKA: protein kinase A; PGE2: prostaglandin E2; PR: progesterone receptor; RT-PCR: reverse transcription polymerase chain reaction; TBS-T: Tris-buffered saline containing 0.1% Tween-20; TNF-α: tumor necrosis factor-alpha; p38MAPK: p38 mitogen-activated protein kinase

## Competing interests

MY, T. Mizuno, and YA are employees of Chugai Pharmaceutical Co. Ltd., Japan. The authors have no other relevant affiliations or financial involvement with any organization or entity with a financial interest in or financial conflict with the subject matter or materials discussed in the manuscript apart from those disclosed.

## Authors' contributions

The work presented here was carried out in collaboration by all the authors. TM and TM defined the study concept. MY and TM designed the methods and experiments, performed the laboratory experiments, analyzed the data, interpreted the results, and wrote the manuscript. TK codesigned the experiments and coworked on the associated data collection and their interpretation. YA codesigned the experiments and participated in the discussion, analyses, interpretation, and presentation of the study data. All authors read and approved the final manuscript.

## Supplementary Material

Additional file 1 **Supplemental Figure S1**. Induction of Prolactin and IGFBP-1 expression in KC02-44D cells. Click here for file

Additional file 2 **Supplemental Figure S2**. Long-term maintenance of KC02-44D cells Click here for file
