# Supplementary material for: Establishment of an immortalized human endometrial stromal cell line with functional responses to ovarian stimuli
Source: Reprod Biol Endocrinol. 2011 Aug 1;9:104. doi: 10.1186/1477-7827-9-104 (PMC3160358; doi:10.1186/1477-7827-9-104)
Supplement: Additional file 1 — Supplemental Figure S1. Induction of Prolactin and IGFBP-1 expression in KC02-44D cells. [file 1477-7827-9-104-S1.PPT]

## Slide 1
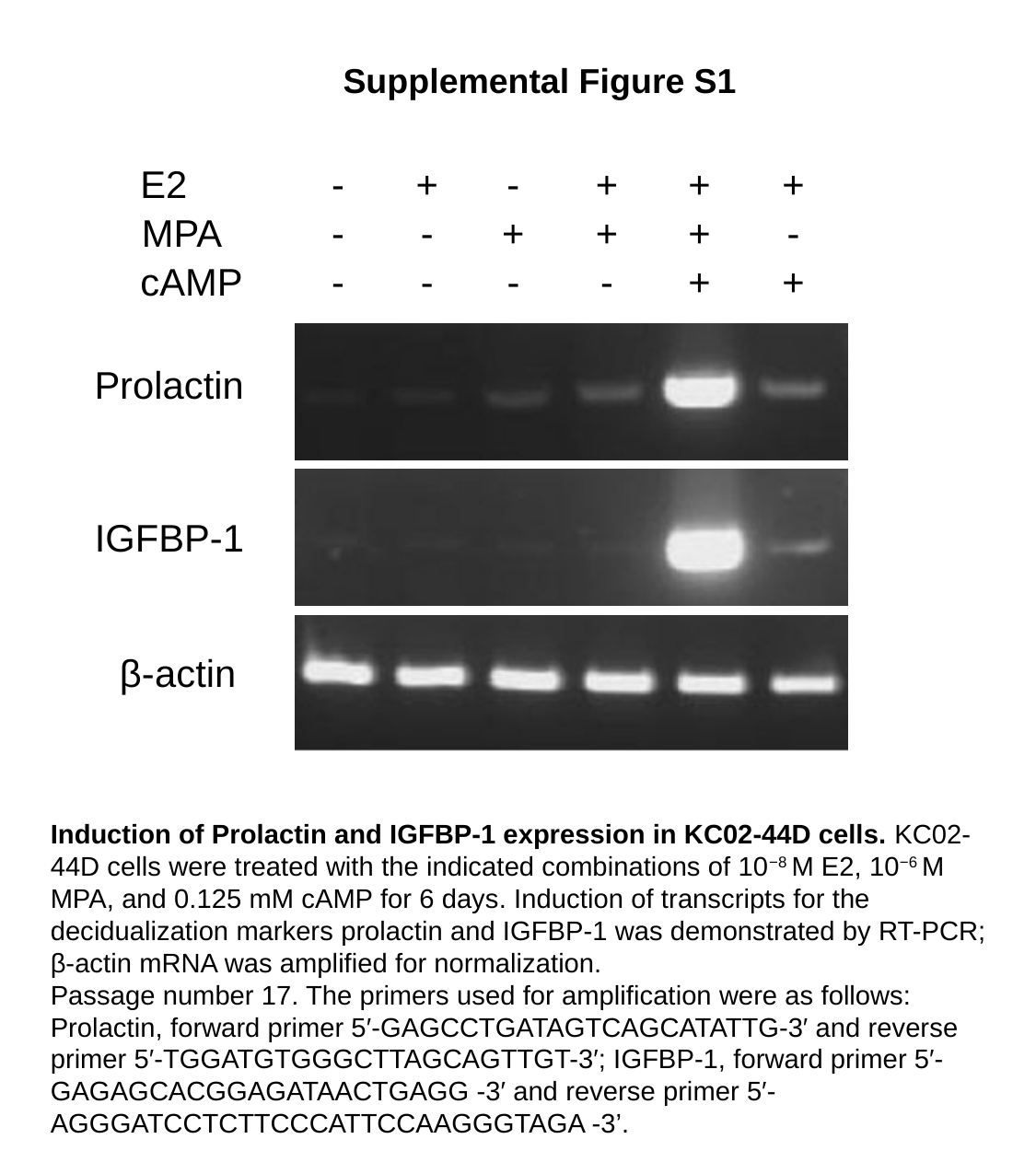

Supplemental Figure S1
E2
-
+
-
+
+
+
MPA
-
-
+
+
+
-
cAMP
-
-
-
-
+
+
Prolactin
IGFBP-1
β-actin
Induction of Prolactin and IGFBP-1 expression in KC02-44D cells. KC02-44D cells were treated with the indicated combinations of 10−8 M E2, 10−6 M MPA, and 0.125 mM cAMP for 6 days. Induction of transcripts for the decidualization markers prolactin and IGFBP-1 was demonstrated by RT-PCR; β-actin mRNA was amplified for normalization.
Passage number 17. The primers used for amplification were as follows: Prolactin, forward primer 5′-GAGCCTGATAGTCAGCATATTG-3′ and reverse primer 5′-TGGATGTGGGCTTAGCAGTTGT-3′; IGFBP-1, forward primer 5′- GAGAGCACGGAGATAACTGAGG -3′ and reverse primer 5′- AGGGATCCTCTTCCCATTCCAAGGGTAGA -3’.
