# Supplementary material for: Establishment of an immortalized human endometrial stromal cell line with functional responses to ovarian stimuli
Source: Reprod Biol Endocrinol. 2011 Aug 1;9:104. doi: 10.1186/1477-7827-9-104 (PMC3160358; doi:10.1186/1477-7827-9-104)
Supplement: Additional file 2 — Supplemental Figure S2. Long-term maintenance of KC02-44D cells [file 1477-7827-9-104-S2.PPT]

## Slide 1
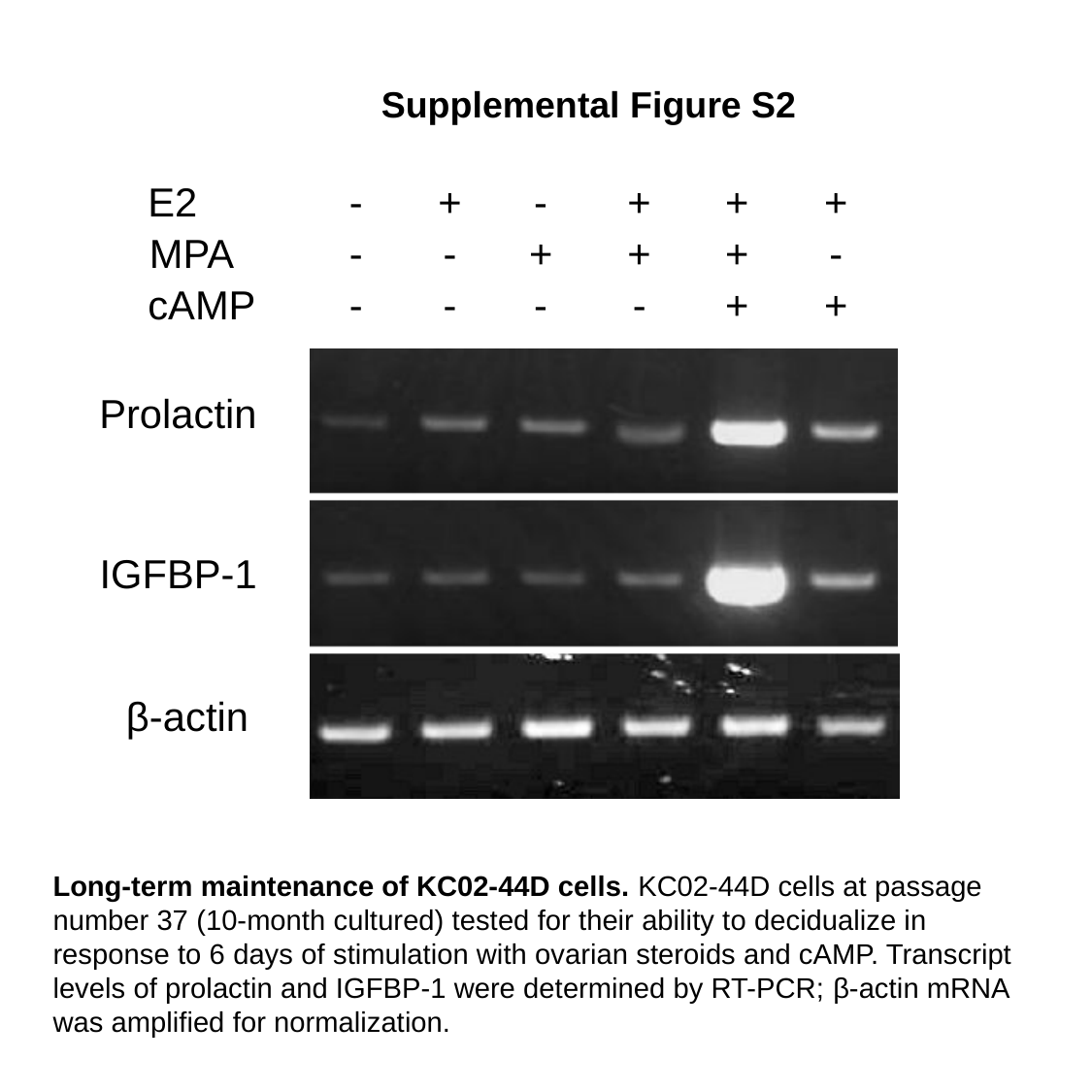

Supplemental Figure S2
E2
-
+
-
+
+
+
MPA
-
-
+
+
+
-
cAMP
-
-
-
-
+
+
Prolactin
IGFBP-1
β-actin
Long-term maintenance of KC02-44D cells. KC02-44D cells at passage number 37 (10-month cultured) tested for their ability to decidualize in response to 6 days of stimulation with ovarian steroids and cAMP. Transcript levels of prolactin and IGFBP-1 were determined by RT-PCR; β-actin mRNA was amplified for normalization.
